# Supplementary material for: Preferential ice growth on grooved surface for crisscross-aligned graphene aerogel with large negative Poisson’s ratio
Source: Nat Commun. 2023 Nov 29;14:7855. doi: 10.1038/s41467-023-43441-6 (PMC10687255; doi:10.1038/s41467-023-43441-6)
Supplement: Supplementary file 1 — Supplementary information [file 41467_2023_43441_MOESM1_ESM.pdf]

# Supplementary Information

## Preferential ice growth on grooved surface for crisscross-aligned graphene aerogel with large negative Poisson's ratio

Meng Li,[1,2] Nifang Zhao,[1] Anran Mao,[1] Mengning Wang,[1] Ziyu Shao,[1] Weiwei Gao[3,*] and Hao Bai[1,2,*]

[1]State Key Laboratory of Chemical Engineering, College of Chemical and Biological Engineering, Zhejiang University, Hangzhou 310058, China

[2]Institute of Zhejiang University-Quzhou, Quzhou 324000, China

[3]Department of Polymer Science and Engineering, Zhejiang University, Hangzhou 310058, China

*Corresponding author. E-mail: Hao Bai (hbai@zju.edu.cn) and Weiwei Gao (wwgao@zju.edu.cn)

**This PDF files includes:**

Supplementary Figs. 1 to 13

Supplementary Tables 1 to 3

**Other Supplementary Materials for this manuscript include the following:**

Supplementary Movies 1 to 3 (.mov)

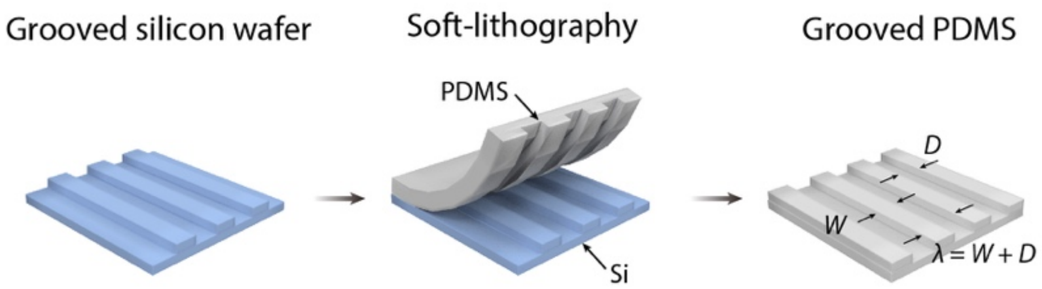


**Supplementary Figure 1 | Scheme showing the fabrication process of the grooved PDMS through soft-lithography.**

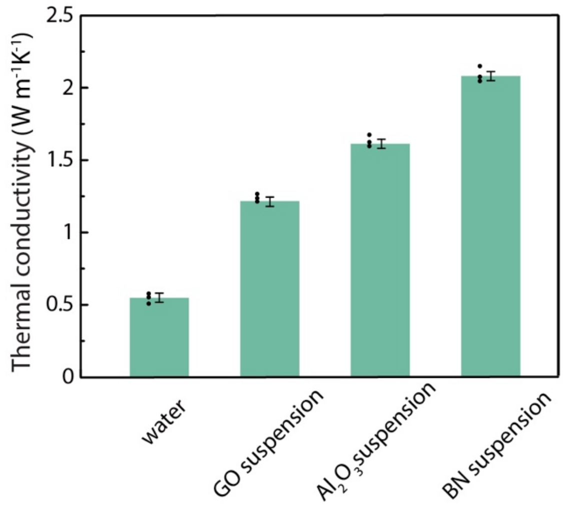


**Supplementary Figure 2 | Thermal conductivity of water and the suspensions with various materials.** The thermal conductivity $k$ was calculated according to the equation $k = \alpha\rho C$, where $\alpha$, $\rho$, and $C$ are the thermal diffusivity, density, and specific heat capacity, respectively. The thermal diffusivity $\alpha$ was measured through the Hot Disk TPS 2500 S in the transient isotropic mode. The errors represent the standard deviations from at least three independent experiments. The black circles show the individual data from every measurement of each sample.

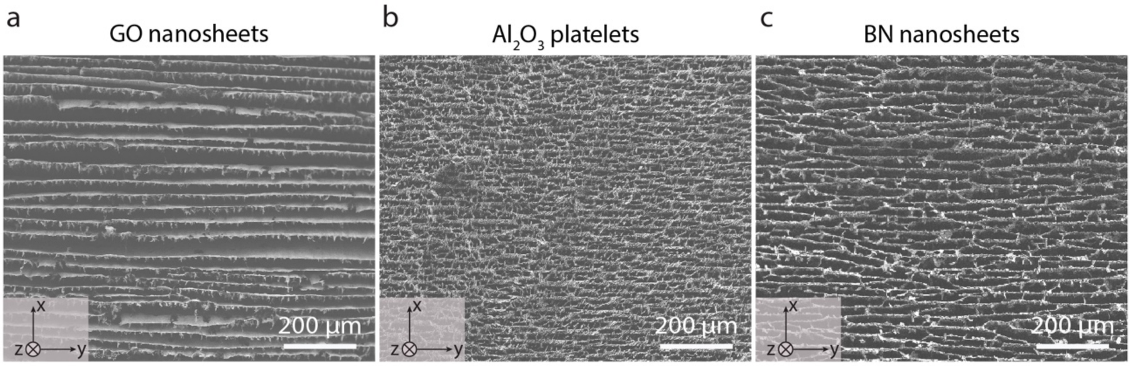


**Supplementary Figure 3 | SEM images of large-area lamellar structures obtained on the grooved surfaces with *W* = *D* = 10 µm with graphene oxide (GO) nanosheets (a), alumina ($Al_2O_3$) platelets (b), and boron nitride (BN) nanosheets (c), respectively.**

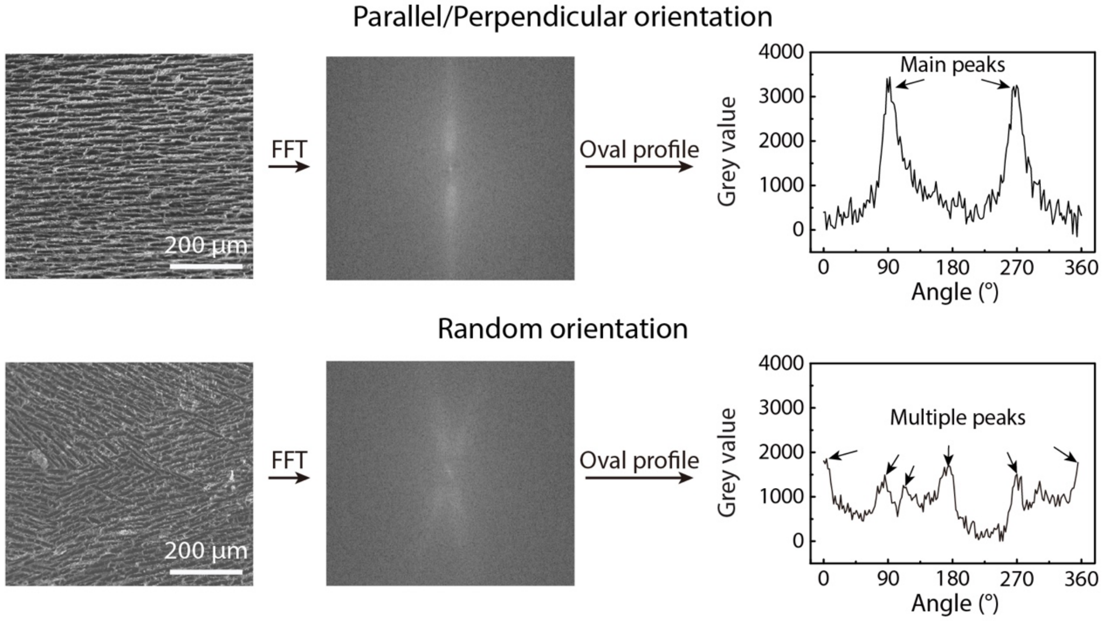


**Supplementary Figure 4 | Processing of the SEM images of the porous architectures indicating aligned (parallel or perpendicular) and random orientation.** Specifically, SEM images were transformed through Fast Fourier Transform (FFT) in Image J, and the intensity distribution was analyzed through the oval profile plugin and plotted as the grey value-angle curve. Grey value represents the number of the grey pixel along a certain direction in the intensity map.

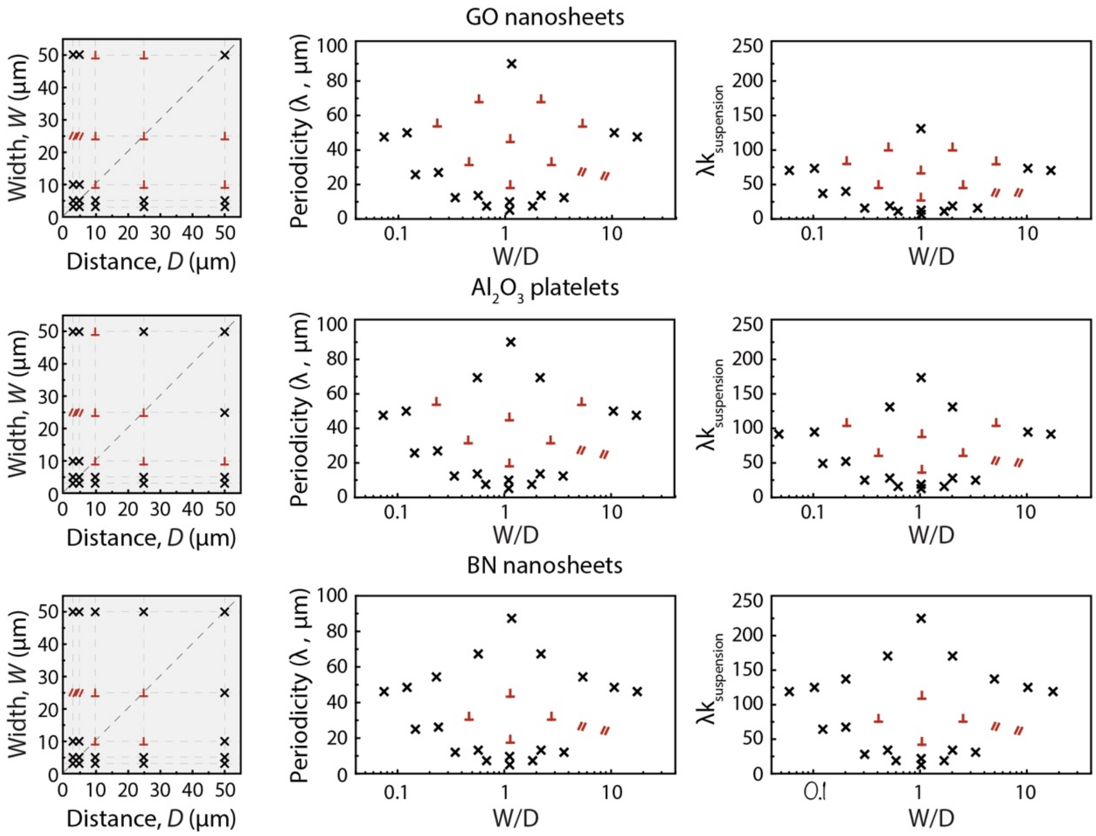


**Supplementary Figure 5 | Three occasions of ice crystals alignment are labelled in the *W-D* map, *λ-W/D* map, and $\lambda k_{suspension}$-*W/D* map for the suspensions of graphene oxide (GO) nanosheets, alumina ($Al_2O_3$) platelets, and boron nitride (BN) nanosheets.**

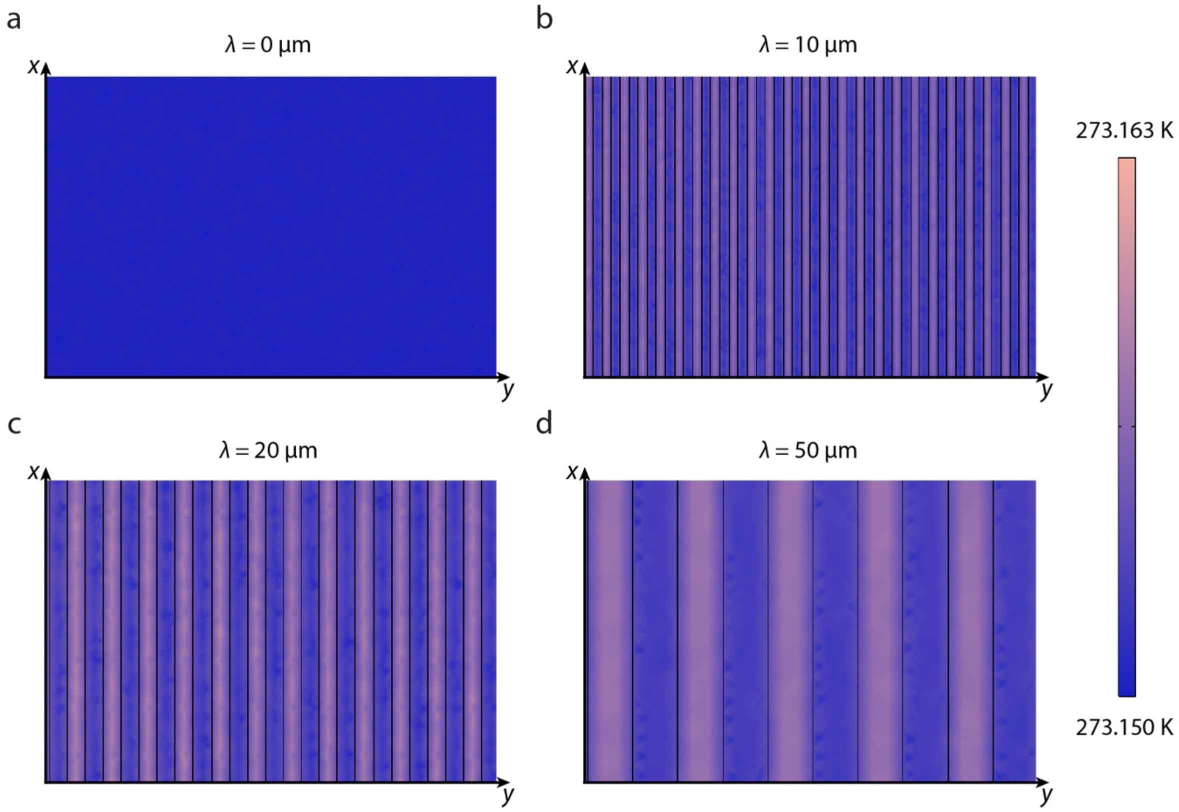


**Supplementary Figure 6 | The temperature distribution at xy plane when it is 0** °C **at the top of the grooves (*z* = *H*). a** On the smooth surface ($\lambda = 0$ μm), the temperature distribution is homogeneous. **b–d** On the grooved surface, the localized temperature gradient is obviously generated along the direction perpendicular to the grooves, which is depending on the groove size.

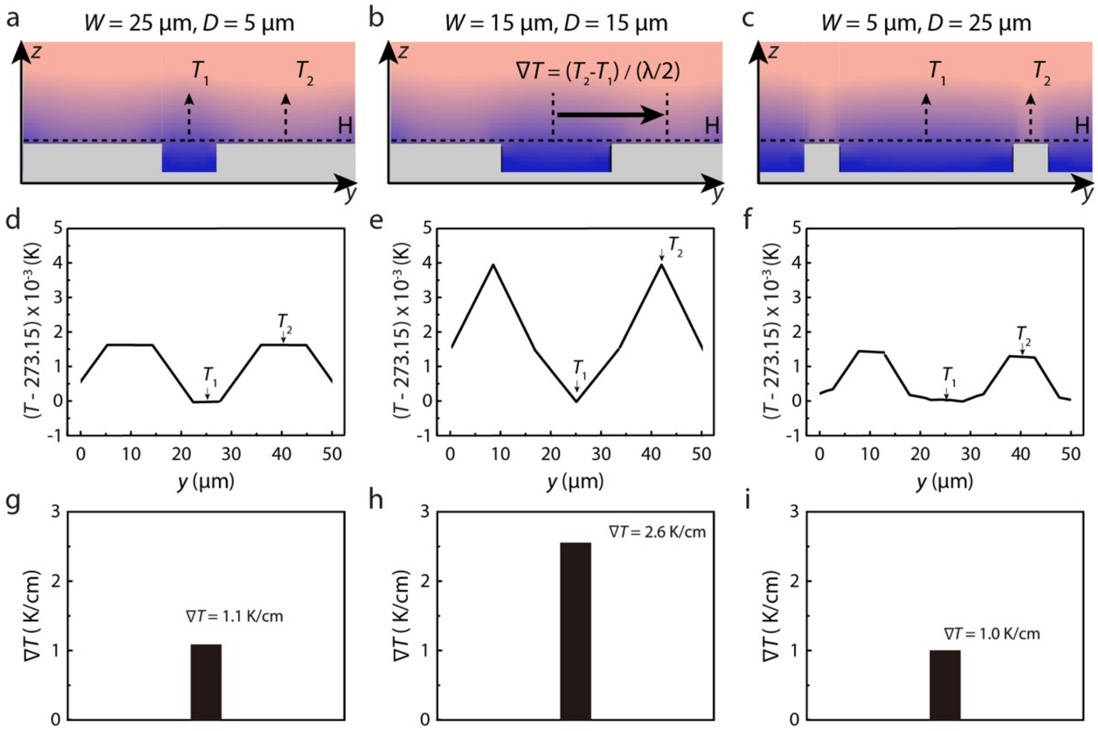


**Supplementary Figure 7 | Simulation analysis of temperature distribution on the grooved surfaces with a constant $\lambda$ (30 μm) and different *W/D*. a-c** Schemes of the theoretical simulation showing the typical temperature distribution on grooved surfaces with $\lambda = 30$ μm and $W/D = 5$, 1, and 0.2, respectively. The blue color represents low temperature and the red color represents high temperature. $T_1$ and $T_2$ are the temperatures at the center of the concave and convex parts at $z = H$. **d-f** Temperature distribution along $y$ position on different grooved surfaces when ice crystals grow up to the top position of the concave parts. **g-i** The localized temperature gradient ($\nabla T = (T_2 - T_1)/(\lambda/2)$) on different grooved surfaces.

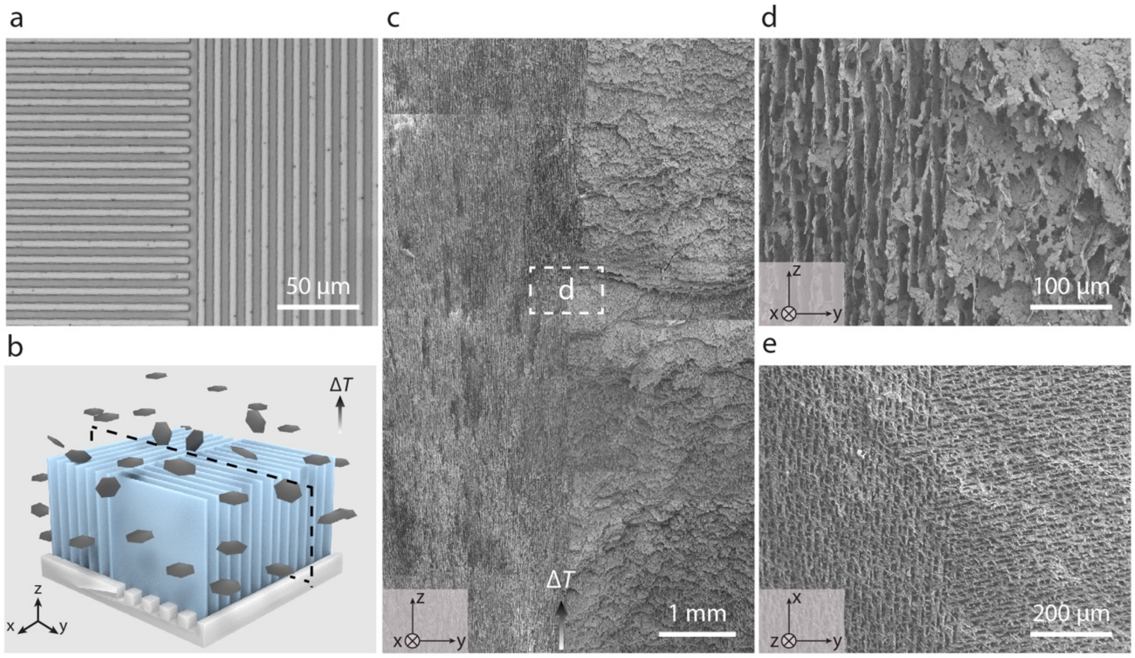


**Supplementary Figure 8 | The designability of the freeze-casting process on the grooved surface.** **a** Optical image of the cross-aligned grooves. **b** Schematic illustration of the directional freezing process on a surface with cross-aligned grooves. **c** SEM image of the microstructure along the temperature gradient direction. **d** SEM image of the enlarged area as labeled in (c). **e** SEM image of the microstructure perpendicular to the temperature gradient direction.

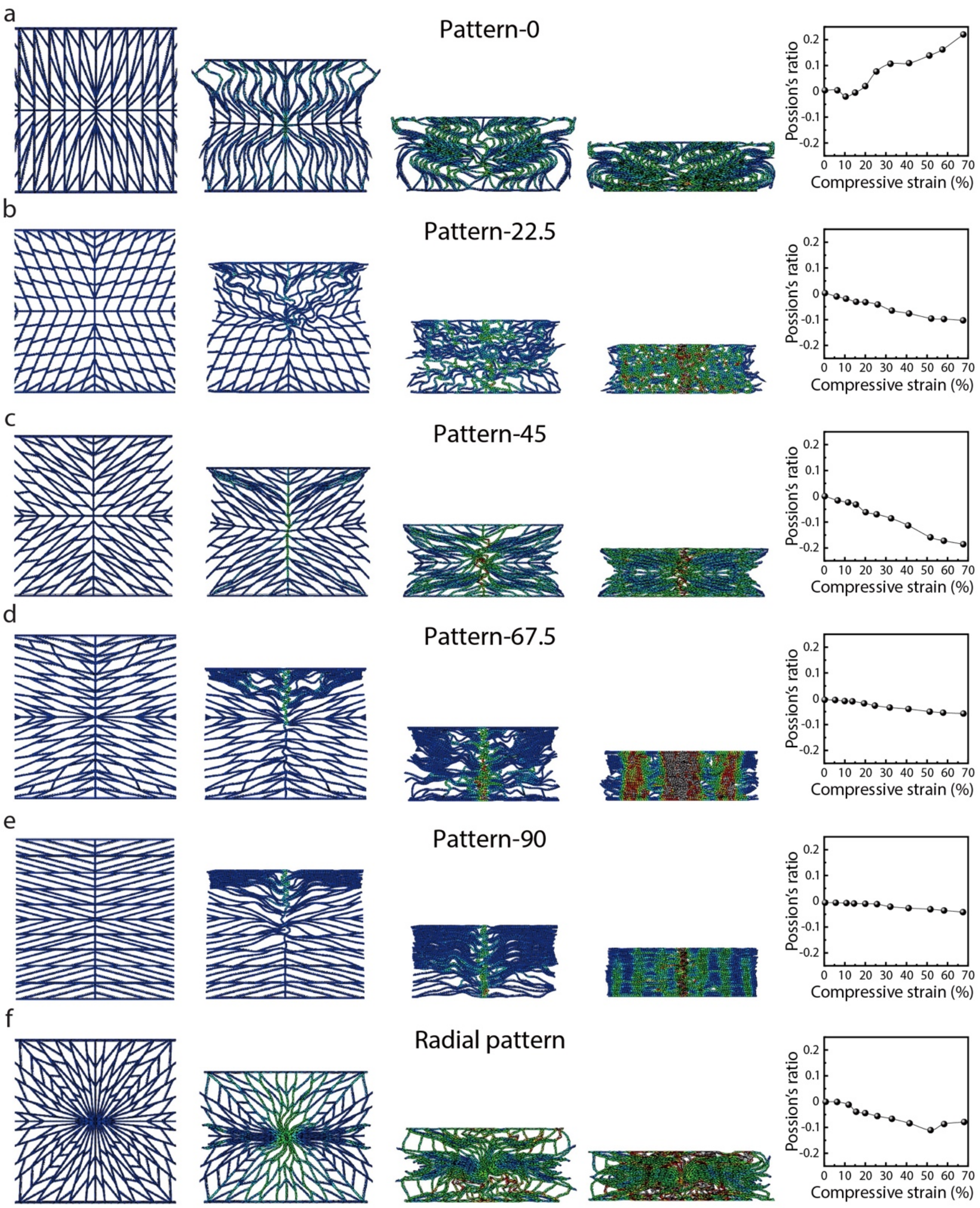


**Supplementary Figure 9 | a-f Finite element simulation of compression processes and the evolution of the corresponding Poisson's ratio as a function of the compressive strain for Pattern-0, Pattern-22.5, Pattern-45, Pattern-67.5, Pattern-90, and Radial pattern, respectively. In each panel, the strain of the structure is 0%, 20%, 54%, and 70%, respectively.**

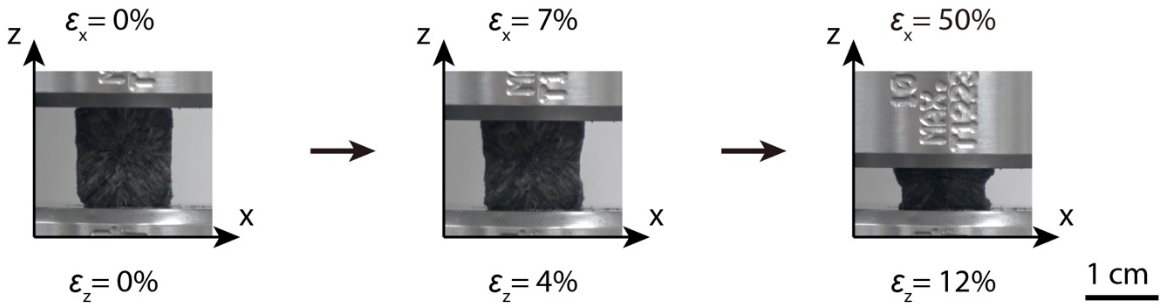


**Supplementary Figure 10 | Optical images of the compression process of crisscross-aligned graphene aerogels.**

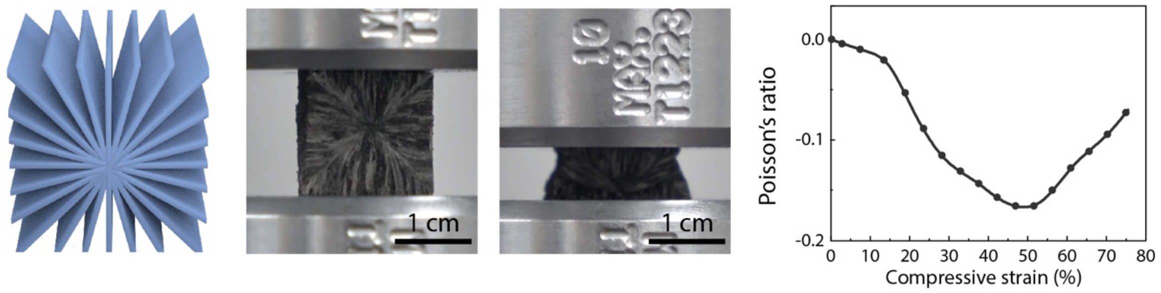


**Supplementary Figure 11 | Optical images of the compression process of radial-aligned graphene aerogels and the evolution of the Poisson's ratio as a function of the compressive strain.**

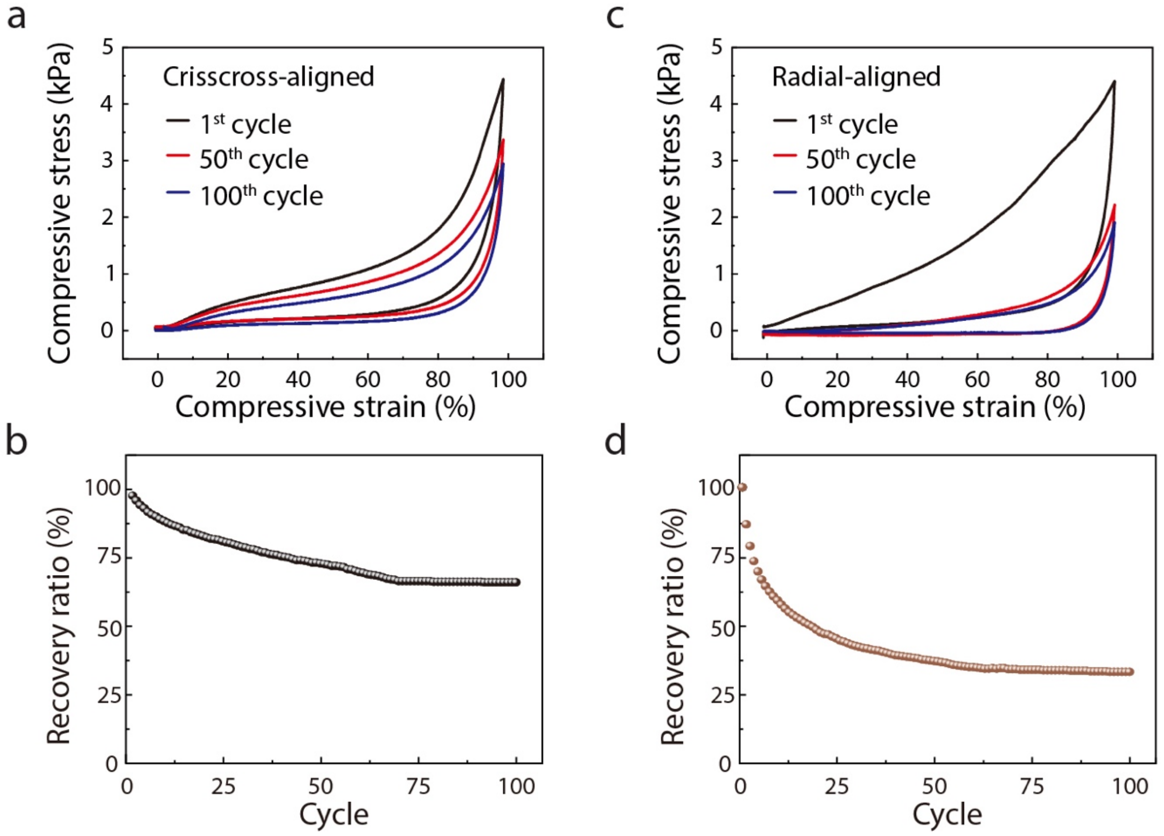


**Supplementary Figure 12 | Compression cycles of graphene aerogels with different architectures.** **a** Plot of the stress and strain of the crisscross-aligned graphene aerogels when undergo compression cycles. **b** Plot of the stress and strain of the radial-aligned graphene aerogels when undergo compression cycles. **c** Plot of the recovery ratio of the stress of the crisscross-aligned graphene aerogels. **d** Plot of the recovery ratio of the stress of the radial-aligned graphene aerogels.

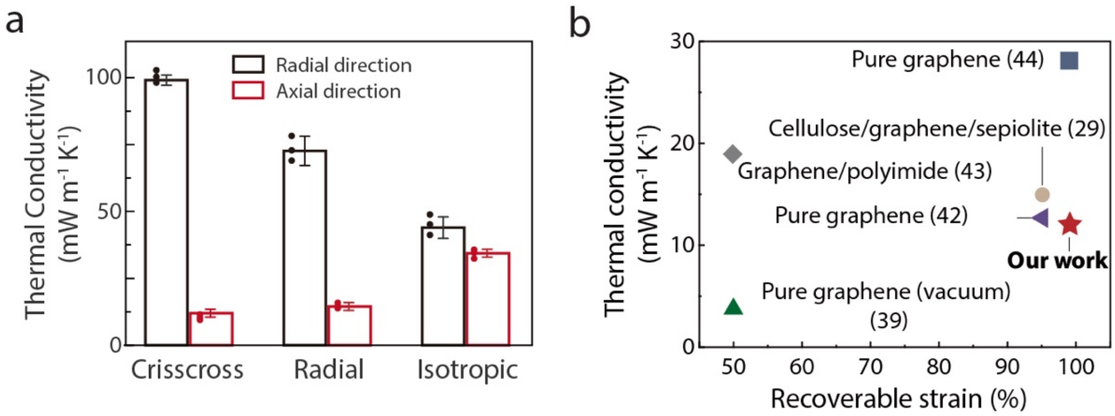


**Supplementary Figure 13 | Thermal property of graphene aerogels. a** Thermal conductivity of three graphene aerogels with different architectures. The thermal conductivity $k$ was calculated according to the equation $k = \alpha\rho C$, where $\alpha$, $\rho$, and $C$ are the thermal diffusivity, density, and specific heat capacity, respectively. The thermal diffusivity $\alpha$ was measured through the Hot Disk TPS 2500 S in the transient anisotropic mode. The errors represent the standard deviations from at least three independent experiments. The circles show the individual data from every measurement of each sample. **b** Ashby map of the thermal conductivity and recoverable strain of various graphene-based aerogels.

**Supplementary Table 1 | The sizes and shapes of the suspended graphene oxide (GO) nanosheets, alumina ($Al_2O_3$) platelets, and boron nitride (BN) nanosheets**

| Medium | Shape | Thickness (nm) |
|---|---|---|
| GO | Nanosheet | 0.8 - 1.2 |
| Alumina | Platelet | ~250 |
| BN | Nanosheet | ~30 |

**Supplementary Table 2 | A summary of fabrication processes and negative Poisson's ratio of graphene-based cellular materials.**

| References | Methods | Microstructures | Poisson's ratio | Density (mg $cm^{-3}$) |
|---|---|---|---|---|
| 36 | Freeze-casting and freeze-drying | Hyperbolic | -0.38 | 8 |
| 37 | Freeze-casting and natural drying | Core-shell-like | -0.3 | 6 |
| 38 | Freeze-casting and freeze-drying | Centripetal | -0.18 | 8 |
| 39 | Freeze-casting and freeze-drying | Twin-structured | -0.2 | 5 |
| 40 | Freeze-casting and freeze-drying | Disordered | Near zero | 2 |
| 41 | Freeze-casting and freeze-drying | Disordered | -0.34 | 5 |
| Our work | Freeze-casting and freeze-drying | crisscross-aligned | -0.55 | 3 |

**Supplementary Table 3 | A summary of recoverable strain and thermal conductivity of graphene-based cellular materials.**

| References | Component | Density (mg $cm^{-3}$) | Recoverable strain (%) | Thermal conductivity (mW $m^{-1}$ $K^{-1}$) |
|---|---|---|---|---|
| 42 | Pure graphene | 2 | 95 | 12.6 (air) |
| 43 | Graphene/polyimide | 9.2 | 50 | 19 (air) |
| 39 | Pure graphene | 5 | 50 | 4.78 (vacuum) |
| 29 | Cellulose/graphene/ sepiolite | 7 | 95 | 15 (air) |
| 44 | Pure graphene | 22.4 | 99 | 28.1 (air) |
| Our work | Pure graphene | 3 | 99 | 12.1 ± 0.2 (air) |
