# Supplementary material for: Preferential ice growth on grooved surface for crisscross-aligned graphene aerogel with large negative Poisson’s ratio
Source: Nat Commun. 2023 Nov 29;14:7855. doi: 10.1038/s41467-023-43441-6 (PMC10687255; doi:10.1038/s41467-023-43441-6)
Supplement: Supplementary file 3 — Description of Additional Supplementary Files [file 41467_2023_43441_MOESM3_ESM.pdf]

Description of Additional Supplementary Files

Supplementary Movie 1: Freezing process on the grooved surface.

Supplementary Movie 2: Finite element simulation of the compression process of porous models with different architectures.

Supplementary Movie 3: Compression process of graphene aerogels with crisscross-aligned and radial-aligned architectures.
